# Supplementary material for: Understanding how individualised physiotherapy or advice altered different elements of disability for people with low back pain using network analysis
Source: PLoS One. 2022 Feb 10;17(2):e0263574. doi: 10.1371/journal.pone.0263574 (PMC8830646; doi:10.1371/journal.pone.0263574)
Supplement: S1 File — (ZIP) [file pone.0263574.s001.zip › supporting/sm_table1.docx]

Table S1. Items and scorings of the Oswestry Disability Index

| Node | Variable | 0 | 1 | 2 | 3 | 4 | 5 |
| --- | --- | --- | --- | --- | --- | --- | --- |
| Grp | Treatment group | Advice | Individualized PT |  | | | |
| Q1 | Pain intensity | I have no pain at the moment | The pain is very mild at the moment | The pain is moderate at the moment | The pain is fairly severe at the moment | The pain is very severe at the moment | The pain is the worst imaginable at the moment |
| Q2 | Personal care (washing, dressing, etc.) | I can look after myself normally without causing extra pain | I can look after myself normally but it causes extra pain | It is painful to look after myself and I am slow and careful | I need some help but can manage most of my personal care | I need help every day in most aspects of self-care | I do not get dressed, wash with difficulty and stay in bed |
| Q3 | Lifting | I can lift heavy weights without extra pain | I can lift heavy weights but it gives me extra pain | Pain prevents me lifting heavy weights off the floor but I can manage if they are conveniently placed e.g. on a table | Pain prevents me lifting heavy weights but I can manage light to medium weights if they are conveniently positioned | I can only lift very light weights | I cannot lift or carry anything |
| Q4 | Walking | Pain does not prevent me walking any distance | Pain prevents me from walking more than 2 kilometres | Pain prevents me from walking more than 1 kilometre | Pain prevents me from walking more than 500 metres | I can only walk using a stick or crutches | I am in bed most of the time |
| Q5 | Sitting | I can sit in any chair as long as I like | I can only sit in my favourite chair as long as I like | Pain prevents me sitting more than one hour | Pain prevents me from sitting more than 30 minutes | Pain prevents me from sitting more than 10 minutes | Pain prevents me from sitting at all |
| Q6 | Standing | I can stand as long as I want without extra pain | I can stand as long as I want but it gives me extra pain | Pain prevents me from standing for more than 1 hour | Pain prevents me from standing for more than 30 minutes | Pain prevents me from standing for more than 10 minutes | Pain prevents me from standing at all |
| Q7 | Sleeping | My sleep is never disturbed by pain | My sleep is occasionally disturbed by pain | Because of pain I have less than 6 hours sleep | Because of pain I have less than 4 hours sleep | Because of pain I have less than 2 hours sleep | Pain prevents me from sleeping at all |
| Q8 | Social life | My social life is normal and gives me no extra pain | My social life is normal but increases the degree of pain | Pain has no significant effect on my social life apart from limiting my more energetic interests e.g. sport | Pain has restricted my social life and I do not go out as often | Pain has restricted my social life to my home | I have no social life because of pain |
| Q9 | Traveling | I can travel anywhere without pain | I can travel anywhere but it gives me extra pain | Pain is bad but I manage journeys over two hours | Pain restricts me to journeys of less than one hour | Pain restricts me to short necessary journeys under 30 minutes | Pain prevents me from traveling except to receive treatment |
| Q10 | Work/Housework | My normal housework/work activities do not cause pain | My normal housework/work activities increase my pain, but I can still perform all that is required of me | I can perform most of my housework/work activities, but pain prevents me from performing more physically stressful activities (eg lifting, vacuuming) | Pain prevents me from doing anything but light housework/work duties | Pain prevents me from doing even light housework/work duties | Pain prevents me from performing any housework/work duties. |
