# Supplementary material for: Understanding how individualised physiotherapy or advice altered different elements of disability for people with low back pain using network analysis
Source: PLoS One. 2022 Feb 10;17(2):e0263574. doi: 10.1371/journal.pone.0263574 (PMC8830646; doi:10.1371/journal.pone.0263574)
Supplement: S1 File — (ZIP) [file pone.0263574.s001.zip › supporting/sm_table2.docx]

Table S2. Edge weights and variability

| Time | Edge | Association | 2.5% | 97.5% |
| --- | --- | --- | --- | --- |
| Baseline | grp--Q1 | 0.000 | -0.140 | 0.079 |
| Baseline | grp--Q10 | 0.000 | -0.126 | 0.082 |
| Baseline | grp--Q2 | 0.000 | -0.074 | 0.133 |
| Baseline | grp--Q3 | 0.000 | -0.115 | 0.115 |
| Baseline | grp--Q4 | 0.000 | -0.157 | 0.000 |
| Baseline | grp--Q5 | 0.000 | 0.000 | 0.176 |
| Baseline | grp--Q6 | -0.087 | -0.290 | 0.000 |
| Baseline | grp--Q7 | 0.000 | -0.108 | 0.121 |
| Baseline | grp--Q8 | 0.000 | -0.090 | 0.124 |
| Baseline | grp--Q9 | 0.000 | 0.000 | 0.210 |
| Baseline | Q1--Q10 | 0.048 | 0.000 | 0.183 |
| Baseline | Q1--Q2 | 0.170 | 0.054 | 0.289 |
| Baseline | Q1--Q3 | 0.000 | 0.000 | 0.143 |
| Baseline | Q1--Q4 | 0.000 | -0.163 | 0.000 |
| Baseline | Q1--Q5 | 0.000 | -0.086 | 0.098 |
| Baseline | Q1--Q6 | 0.000 | 0.000 | 0.155 |
| Baseline | Q1--Q7 | 0.138 | 0.000 | 0.263 |
| Baseline | Q1--Q8 | 0.000 | -0.066 | 0.116 |
| Baseline | Q1--Q9 | 0.151 | 0.000 | 0.268 |
| Baseline | Q2--Q10 | 0.147 | 0.000 | 0.252 |
| Baseline | Q2--Q3 | 0.079 | 0.000 | 0.190 |
| Baseline | Q2--Q4 | 0.000 | -0.055 | 0.106 |
| Baseline | Q2--Q5 | 0.106 | 0.000 | 0.218 |
| Baseline | Q2--Q6 | 0.000 | -0.153 | 0.000 |
| Baseline | Q2--Q7 | 0.000 | 0.000 | 0.166 |
| Baseline | Q2--Q8 | 0.173 | 0.058 | 0.282 |
| Baseline | Q2--Q9 | 0.113 | 0.000 | 0.232 |
| Baseline | Q3--Q10 | 0.300 | 0.188 | 0.400 |
| Baseline | Q3--Q4 | 0.048 | 0.000 | 0.173 |
| Baseline | Q3--Q5 | 0.000 | 0.000 | 0.158 |
| Baseline | Q3--Q6 | 0.096 | 0.000 | 0.209 |
| Baseline | Q3--Q7 | 0.000 | 0.000 | 0.115 |
| Baseline | Q3--Q8 | 0.000 | 0.000 | 0.124 |
| Baseline | Q3--Q9 | 0.000 | -0.077 | 0.099 |
| Baseline | Q4--Q10 | 0.112 | 0.000 | 0.226 |
| Baseline | Q4--Q5 | 0.000 | 0.000 | 0.129 |
| Baseline | Q4--Q6 | 0.323 | 0.216 | 0.444 |
| Baseline | Q4--Q7 | 0.000 | 0.000 | 0.169 |
| Baseline | Q4--Q8 | 0.224 | 0.110 | 0.325 |
| Baseline | Q4--Q9 | 0.000 | -0.065 | 0.121 |
| Baseline | Q5--Q10 | 0.000 | -0.077 | 0.079 |
| Baseline | Q5--Q6 | 0.000 | -0.124 | 0.000 |
| Baseline | Q5--Q7 | 0.000 | 0.000 | 0.150 |
| Baseline | Q5--Q8 | 0.000 | -0.099 | 0.055 |
| Baseline | Q5--Q9 | 0.338 | 0.246 | 0.455 |
| Baseline | Q6--Q10 | 0.073 | 0.000 | 0.191 |
| Baseline | Q6--Q7 | 0.000 | -0.052 | 0.119 |
| Baseline | Q6--Q8 | 0.082 | 0.000 | 0.195 |
| Baseline | Q6--Q9 | 0.000 | -0.070 | 0.096 |
| Baseline | Q7--Q10 | 0.055 | 0.000 | 0.173 |
| Baseline | Q7--Q8 | 0.000 | 0.000 | 0.138 |
| Baseline | Q7--Q9 | 0.000 | -0.153 | 0.051 |
| Baseline | Q8--Q10 | 0.150 | 0.000 | 0.260 |
| Baseline | Q8--Q9 | 0.182 | 0.064 | 0.298 |
| Baseline | Q9--Q10 | 0.184 | 0.072 | 0.288 |
| Baseline | grp | 0.087 | 0.000 | 0.823 |
| Baseline | Q1 | 0.506 | 0.431 | 1.065 |
| Baseline | Q10 | 1.069 | 0.866 | 1.342 |
| Baseline | Q2 | 0.788 | 0.667 | 1.210 |
| Baseline | Q3 | 0.523 | 0.480 | 0.967 |
| Baseline | Q4 | 0.707 | 0.654 | 1.206 |
| Baseline | Q5 | 0.444 | 0.406 | 1.031 |
| Baseline | Q6 | 0.662 | 0.502 | 1.201 |
| Baseline | Q7 | 0.193 | 0.201 | 0.852 |
| Baseline | Q8 | 0.811 | 0.697 | 1.217 |
| Baseline | Q9 | 0.967 | 0.854 | 1.451 |
| Week 5 | grp--Q1 | -0.143 | -0.338 | 0.000 |
| Week 5 | grp--Q10 | 0.000 | -0.186 | 0.076 |
| Week 5 | grp--Q2 | 0.000 | -0.198 | 0.063 |
| Week 5 | grp--Q3 | 0.156 | 0.000 | 0.350 |
| Week 5 | grp--Q4 | 0.000 | -0.118 | 0.149 |
| Week 5 | grp--Q5 | 0.000 | -0.091 | 0.169 |
| Week 5 | grp--Q6 | 0.000 | -0.225 | 0.056 |
| Week 5 | grp--Q7 | -0.241 | -0.417 | -0.068 |
| Week 5 | grp--Q8 | 0.000 | 0.000 | 0.253 |
| Week 5 | grp--Q9 | 0.078 | 0.000 | 0.266 |
| Week 5 | Q1--Q10 | 0.000 | 0.000 | 0.133 |
| Week 5 | Q1--Q2 | 0.195 | 0.070 | 0.302 |
| Week 5 | Q1--Q3 | 0.000 | -0.116 | 0.084 |
| Week 5 | Q1--Q4 | 0.084 | 0.000 | 0.192 |
| Week 5 | Q1--Q5 | 0.000 | -0.089 | 0.108 |
| Week 5 | Q1--Q6 | 0.117 | 0.000 | 0.233 |
| Week 5 | Q1--Q7 | 0.180 | 0.056 | 0.282 |
| Week 5 | Q1--Q8 | 0.000 | -0.120 | 0.126 |
| Week 5 | Q1--Q9 | 0.206 | 0.084 | 0.314 |
| Week 5 | Q2--Q10 | 0.073 | 0.000 | 0.170 |
| Week 5 | Q2--Q3 | 0.042 | 0.000 | 0.145 |
| Week 5 | Q2--Q4 | 0.041 | 0.000 | 0.181 |
| Week 5 | Q2--Q5 | 0.000 | -0.085 | 0.099 |
| Week 5 | Q2--Q6 | 0.000 | -0.085 | 0.100 |
| Week 5 | Q2--Q7 | 0.062 | 0.000 | 0.185 |
| Week 5 | Q2--Q8 | 0.204 | 0.077 | 0.328 |
| Week 5 | Q2--Q9 | 0.000 | 0.000 | 0.179 |
| Week 5 | Q3--Q10 | 0.292 | 0.168 | 0.408 |
| Week 5 | Q3--Q4 | 0.079 | 0.000 | 0.185 |
| Week 5 | Q3--Q5 | 0.000 | -0.106 | 0.083 |
| Week 5 | Q3--Q6 | 0.000 | -0.054 | 0.130 |
| Week 5 | Q3--Q7 | 0.000 | -0.083 | 0.067 |
| Week 5 | Q3--Q8 | 0.099 | 0.000 | 0.202 |
| Week 5 | Q3--Q9 | 0.054 | 0.000 | 0.169 |
| Week 5 | Q4--Q10 | 0.103 | 0.000 | 0.211 |
| Week 5 | Q4--Q5 | 0.000 | 0.000 | 0.148 |
| Week 5 | Q4--Q6 | 0.244 | 0.124 | 0.358 |
| Week 5 | Q4--Q7 | 0.000 | 0.000 | 0.139 |
| Week 5 | Q4--Q8 | 0.135 | 0.000 | 0.266 |
| Week 5 | Q4--Q9 | 0.000 | -0.119 | 0.088 |
| Week 5 | Q5--Q10 | 0.000 | 0.000 | 0.148 |
| Week 5 | Q5--Q6 | 0.056 | 0.000 | 0.186 |
| Week 5 | Q5--Q7 | 0.108 | 0.000 | 0.210 |
| Week 5 | Q5--Q8 | 0.000 | -0.169 | 0.000 |
| Week 5 | Q5--Q9 | 0.408 | 0.296 | 0.502 |
| Week 5 | Q6--Q10 | 0.000 | 0.000 | 0.139 |
| Week 5 | Q6--Q7 | 0.000 | -0.071 | 0.138 |
| Week 5 | Q6--Q8 | 0.168 | 0.000 | 0.281 |
| Week 5 | Q6--Q9 | 0.000 | -0.086 | 0.113 |
| Week 5 | Q7--Q10 | 0.165 | 0.000 | 0.268 |
| Week 5 | Q7--Q8 | 0.000 | 0.000 | 0.165 |
| Week 5 | Q7--Q9 | 0.000 | 0.000 | 0.156 |
| Week 5 | Q8--Q10 | 0.308 | 0.197 | 0.403 |
| Week 5 | Q8--Q9 | 0.238 | 0.130 | 0.357 |
| Week 5 | Q9--Q10 | 0.083 | 0.000 | 0.192 |
| Week 5 | grp | 0.618 | 0.373 | 1.543 |
| Week 5 | Q1 | 0.925 | 0.699 | 1.368 |
| Week 5 | Q10 | 1.023 | 0.857 | 1.371 |
| Week 5 | Q2 | 0.617 | 0.498 | 1.018 |
| Week 5 | Q3 | 0.722 | 0.503 | 1.114 |
| Week 5 | Q4 | 0.687 | 0.535 | 1.098 |
| Week 5 | Q5 | 0.572 | 0.447 | 1.199 |
| Week 5 | Q6 | 0.586 | 0.495 | 1.137 |
| Week 5 | Q7 | 0.755 | 0.606 | 1.166 |
| Week 5 | Q8 | 1.153 | 1.009 | 1.707 |
| Week 5 | Q9 | 1.067 | 0.901 | 1.492 |
| Week 10 | grp--Q1 | -0.099 | -0.331 | 0.000 |
| Week 10 | grp--Q10 | 0.000 | 0.000 | 0.265 |
| Week 10 | grp--Q2 | 0.000 | -0.130 | 0.134 |
| Week 10 | grp--Q3 | 0.000 | -0.151 | 0.122 |
| Week 10 | grp--Q4 | 0.000 | 0.000 | 0.223 |
| Week 10 | grp--Q5 | 0.000 | 0.000 | 0.259 |
| Week 10 | grp--Q6 | -0.065 | -0.316 | 0.000 |
| Week 10 | grp--Q7 | -0.214 | -0.431 | -0.079 |
| Week 10 | grp--Q8 | 0.000 | -0.093 | 0.180 |
| Week 10 | grp--Q9 | 0.000 | -0.201 | 0.074 |
| Week 10 | Q1--Q10 | 0.108 | 0.000 | 0.232 |
| Week 10 | Q1--Q2 | 0.288 | 0.175 | 0.399 |
| Week 10 | Q1--Q3 | 0.106 | 0.000 | 0.232 |
| Week 10 | Q1--Q4 | 0.000 | -0.190 | 0.000 |
| Week 10 | Q1--Q5 | 0.142 | 0.000 | 0.262 |
| Week 10 | Q1--Q6 | 0.146 | 0.000 | 0.270 |
| Week 10 | Q1--Q7 | 0.110 | 0.000 | 0.229 |
| Week 10 | Q1--Q8 | 0.099 | 0.000 | 0.222 |
| Week 10 | Q1--Q9 | 0.000 | 0.000 | 0.144 |
| Week 10 | Q2--Q10 | 0.090 | 0.000 | 0.199 |
| Week 10 | Q2--Q3 | 0.000 | -0.101 | 0.070 |
| Week 10 | Q2--Q4 | 0.000 | 0.000 | 0.138 |
| Week 10 | Q2--Q5 | 0.000 | -0.093 | 0.104 |
| Week 10 | Q2--Q6 | 0.000 | -0.090 | 0.103 |
| Week 10 | Q2--Q7 | 0.049 | 0.000 | 0.171 |
| Week 10 | Q2--Q8 | 0.083 | 0.000 | 0.196 |
| Week 10 | Q2--Q9 | 0.123 | 0.000 | 0.239 |
| Week 10 | Q3--Q10 | 0.250 | 0.113 | 0.364 |
| Week 10 | Q3--Q4 | 0.062 | 0.000 | 0.198 |
| Week 10 | Q3--Q5 | 0.000 | 0.000 | 0.129 |
| Week 10 | Q3--Q6 | 0.129 | 0.000 | 0.251 |
| Week 10 | Q3--Q7 | 0.000 | -0.166 | 0.000 |
| Week 10 | Q3--Q8 | 0.115 | 0.000 | 0.249 |
| Week 10 | Q3--Q9 | 0.143 | 0.000 | 0.254 |
| Week 10 | Q4--Q10 | 0.043 | 0.000 | 0.177 |
| Week 10 | Q4--Q5 | 0.000 | -0.103 | 0.106 |
| Week 10 | Q4--Q6 | 0.323 | 0.211 | 0.452 |
| Week 10 | Q4--Q7 | 0.000 | 0.000 | 0.210 |
| Week 10 | Q4--Q8 | 0.205 | 0.080 | 0.318 |
| Week 10 | Q4--Q9 | 0.000 | -0.129 | 0.091 |
| Week 10 | Q5--Q10 | 0.049 | 0.000 | 0.173 |
| Week 10 | Q5--Q6 | 0.127 | 0.000 | 0.267 |
| Week 10 | Q5--Q7 | 0.123 | 0.000 | 0.247 |
| Week 10 | Q5--Q8 | 0.000 | -0.124 | 0.086 |
| Week 10 | Q5--Q9 | 0.297 | 0.158 | 0.418 |
| Week 10 | Q6--Q10 | 0.134 | 0.000 | 0.275 |
| Week 10 | Q6--Q7 | 0.000 | -0.107 | 0.084 |
| Week 10 | Q6--Q8 | 0.000 | -0.096 | 0.122 |
| Week 10 | Q6--Q9 | 0.000 | -0.220 | 0.000 |
| Week 10 | Q7--Q10 | 0.186 | 0.071 | 0.297 |
| Week 10 | Q7--Q8 | 0.000 | -0.056 | 0.135 |
| Week 10 | Q7--Q9 | 0.000 | -0.076 | 0.150 |
| Week 10 | Q8--Q10 | 0.201 | 0.066 | 0.318 |
| Week 10 | Q8--Q9 | 0.252 | 0.126 | 0.385 |
| Week 10 | Q9--Q10 | 0.172 | 0.000 | 0.307 |
| Week 10 | grp | 0.378 | 0.243 | 1.595 |
| Week 10 | Q1 | 1.097 | 0.952 | 1.577 |
| Week 10 | Q10 | 1.234 | 1.024 | 1.626 |
| Week 10 | Q2 | 0.634 | 0.487 | 1.039 |
| Week 10 | Q3 | 0.805 | 0.671 | 1.242 |
| Week 10 | Q4 | 0.634 | 0.557 | 1.373 |
| Week 10 | Q5 | 0.738 | 0.621 | 1.286 |
| Week 10 | Q6 | 0.924 | 0.793 | 1.601 |
| Week 10 | Q7 | 0.681 | 0.587 | 1.339 |
| Week 10 | Q8 | 0.955 | 0.795 | 1.378 |
| Week 10 | Q9 | 0.987 | 0.825 | 1.559 |
| Week 26 | grp--Q1 | -0.151 | -0.339 | 0.000 |
| Week 26 | grp--Q10 | 0.000 | -0.129 | 0.150 |
| Week 26 | grp--Q2 | 0.000 | -0.100 | 0.177 |
| Week 26 | grp--Q3 | 0.000 | -0.181 | 0.056 |
| Week 26 | grp--Q4 | 0.000 | -0.220 | 0.000 |
| Week 26 | grp--Q5 | 0.000 | 0.000 | 0.240 |
| Week 26 | grp--Q6 | 0.000 | -0.134 | 0.119 |
| Week 26 | grp--Q7 | -0.158 | -0.365 | 0.000 |
| Week 26 | grp--Q8 | 0.000 | 0.000 | 0.225 |
| Week 26 | grp--Q9 | 0.000 | -0.127 | 0.131 |
| Week 26 | Q1--Q10 | 0.239 | 0.102 | 0.362 |
| Week 26 | Q1--Q2 | 0.205 | 0.071 | 0.307 |
| Week 26 | Q1--Q3 | 0.063 | 0.000 | 0.203 |
| Week 26 | Q1--Q4 | 0.000 | -0.179 | 0.000 |
| Week 26 | Q1--Q5 | 0.000 | 0.000 | 0.187 |
| Week 26 | Q1--Q6 | 0.142 | 0.000 | 0.245 |
| Week 26 | Q1--Q7 | 0.177 | 0.000 | 0.290 |
| Week 26 | Q1--Q8 | 0.123 | 0.000 | 0.238 |
| Week 26 | Q1--Q9 | 0.000 | -0.092 | 0.142 |
| Week 26 | Q2--Q10 | 0.053 | 0.000 | 0.173 |
| Week 26 | Q2--Q3 | 0.000 | 0.000 | 0.126 |
| Week 26 | Q2--Q4 | 0.067 | 0.000 | 0.229 |
| Week 26 | Q2--Q5 | 0.000 | -0.164 | 0.000 |
| Week 26 | Q2--Q6 | 0.000 | 0.000 | 0.148 |
| Week 26 | Q2--Q7 | 0.000 | 0.000 | 0.162 |
| Week 26 | Q2--Q8 | 0.110 | 0.000 | 0.244 |
| Week 26 | Q2--Q9 | 0.186 | 0.076 | 0.298 |
| Week 26 | Q3--Q10 | 0.233 | 0.092 | 0.370 |
| Week 26 | Q3--Q4 | 0.000 | -0.126 | 0.091 |
| Week 26 | Q3--Q5 | 0.095 | 0.000 | 0.236 |
| Week 26 | Q3--Q6 | 0.054 | 0.000 | 0.193 |
| Week 26 | Q3--Q7 | 0.000 | -0.163 | 0.000 |
| Week 26 | Q3--Q8 | 0.112 | 0.000 | 0.237 |
| Week 26 | Q3--Q9 | 0.074 | 0.000 | 0.202 |
| Week 26 | Q4--Q10 | 0.171 | 0.000 | 0.321 |
| Week 26 | Q4--Q5 | 0.000 | -0.136 | 0.087 |
| Week 26 | Q4--Q6 | 0.326 | 0.219 | 0.452 |
| Week 26 | Q4--Q7 | 0.000 | -0.102 | 0.096 |
| Week 26 | Q4--Q8 | 0.232 | 0.126 | 0.368 |
| Week 26 | Q4--Q9 | 0.000 | -0.143 | 0.078 |
| Week 26 | Q5--Q10 | 0.098 | 0.000 | 0.227 |
| Week 26 | Q5--Q6 | 0.112 | 0.000 | 0.251 |
| Week 26 | Q5--Q7 | 0.223 | 0.109 | 0.342 |
| Week 26 | Q5--Q8 | 0.000 | -0.084 | 0.150 |
| Week 26 | Q5--Q9 | 0.368 | 0.244 | 0.485 |
| Week 26 | Q6--Q10 | 0.062 | 0.000 | 0.190 |
| Week 26 | Q6--Q7 | 0.099 | 0.000 | 0.227 |
| Week 26 | Q6--Q8 | 0.000 | -0.134 | 0.102 |
| Week 26 | Q6--Q9 | 0.000 | 0.000 | 0.182 |
| Week 26 | Q7--Q10 | 0.000 | 0.000 | 0.158 |
| Week 26 | Q7--Q8 | 0.000 | -0.080 | 0.132 |
| Week 26 | Q7--Q9 | 0.094 | 0.000 | 0.220 |
| Week 26 | Q8--Q10 | 0.201 | 0.071 | 0.323 |
| Week 26 | Q8--Q9 | 0.147 | 0.000 | 0.267 |
| Week 26 | Q9--Q10 | 0.107 | 0.000 | 0.237 |
| Week 26 | grp | 0.308 | 0.182 | 1.310 |
| Week 26 | Q1 | 1.100 | 0.868 | 1.600 |
| Week 26 | Q10 | 1.164 | 0.980 | 1.473 |
| Week 26 | Q2 | 0.621 | 0.503 | 1.156 |
| Week 26 | Q3 | 0.630 | 0.478 | 1.159 |
| Week 26 | Q4 | 0.794 | 0.665 | 1.494 |
| Week 26 | Q5 | 0.895 | 0.810 | 1.474 |
| Week 26 | Q6 | 0.795 | 0.680 | 1.232 |
| Week 26 | Q7 | 0.751 | 0.610 | 1.275 |
| Week 26 | Q8 | 0.925 | 0.778 | 1.427 |
| Week 26 | Q9 | 0.975 | 0.841 | 1.438 |
| Week52 | grp--Q1 | -0.046 | -0.260 | 0.000 |
| Week52 | grp--Q10 | 0.000 | 0.000 | 0.212 |
| Week52 | grp--Q2 | 0.000 | -0.177 | 0.044 |
| Week52 | grp--Q3 | 0.000 | -0.067 | 0.173 |
| Week52 | grp--Q4 | 0.000 | -0.118 | 0.129 |
| Week52 | grp--Q5 | 0.000 | -0.171 | 0.000 |
| Week52 | grp--Q6 | 0.000 | -0.088 | 0.172 |
| Week52 | grp--Q7 | -0.104 | -0.288 | 0.000 |
| Week52 | grp--Q8 | 0.000 | 0.000 | 0.202 |
| Week52 | grp--Q9 | 0.000 | -0.214 | 0.000 |
| Week52 | Q1--Q10 | 0.207 | 0.069 | 0.328 |
| Week52 | Q1--Q2 | 0.176 | 0.000 | 0.313 |
| Week52 | Q1--Q3 | 0.234 | 0.102 | 0.351 |
| Week52 | Q1--Q4 | 0.063 | 0.000 | 0.190 |
| Week52 | Q1--Q5 | 0.055 | 0.000 | 0.189 |
| Week52 | Q1--Q6 | 0.138 | 0.000 | 0.245 |
| Week52 | Q1--Q7 | 0.074 | 0.000 | 0.194 |
| Week52 | Q1--Q8 | 0.000 | 0.000 | 0.161 |
| Week52 | Q1--Q9 | 0.000 | -0.154 | 0.084 |
| Week52 | Q2--Q10 | 0.057 | 0.000 | 0.167 |
| Week52 | Q2--Q3 | 0.000 | -0.049 | 0.084 |
| Week52 | Q2--Q4 | 0.066 | 0.000 | 0.238 |
| Week52 | Q2--Q5 | 0.000 | -0.186 | 0.000 |
| Week52 | Q2--Q6 | 0.000 | -0.135 | 0.081 |
| Week52 | Q2--Q7 | 0.120 | 0.000 | 0.251 |
| Week52 | Q2--Q8 | 0.225 | 0.000 | 0.372 |
| Week52 | Q2--Q9 | 0.142 | 0.000 | 0.269 |
| Week52 | Q3--Q10 | 0.270 | 0.110 | 0.391 |
| Week52 | Q3--Q4 | 0.059 | 0.000 | 0.178 |
| Week52 | Q3--Q5 | 0.000 | 0.000 | 0.148 |
| Week52 | Q3--Q6 | 0.000 | -0.067 | 0.136 |
| Week52 | Q3--Q7 | 0.000 | -0.105 | 0.080 |
| Week52 | Q3--Q8 | 0.000 | -0.093 | 0.113 |
| Week52 | Q3--Q9 | 0.090 | 0.000 | 0.222 |
| Week52 | Q4--Q10 | 0.059 | 0.000 | 0.198 |
| Week52 | Q4--Q5 | 0.064 | 0.000 | 0.218 |
| Week52 | Q4--Q6 | 0.163 | 0.000 | 0.290 |
| Week52 | Q4--Q7 | 0.072 | 0.000 | 0.197 |
| Week52 | Q4--Q8 | 0.284 | 0.151 | 0.409 |
| Week52 | Q4--Q9 | 0.000 | -0.165 | 0.080 |
| Week52 | Q5--Q10 | 0.097 | 0.000 | 0.246 |
| Week52 | Q5--Q6 | 0.297 | 0.184 | 0.410 |
| Week52 | Q5--Q7 | 0.100 | 0.000 | 0.246 |
| Week52 | Q5--Q8 | 0.000 | -0.170 | 0.000 |
| Week52 | Q5--Q9 | 0.257 | 0.130 | 0.397 |
| Week52 | Q6--Q10 | 0.000 | -0.140 | 0.106 |
| Week52 | Q6--Q7 | 0.071 | 0.000 | 0.199 |
| Week52 | Q6--Q8 | 0.172 | 0.000 | 0.296 |
| Week52 | Q6--Q9 | 0.051 | 0.000 | 0.195 |
| Week52 | Q7--Q10 | 0.078 | 0.000 | 0.200 |
| Week52 | Q7--Q8 | 0.000 | 0.000 | 0.140 |
| Week52 | Q7--Q9 | 0.131 | 0.000 | 0.270 |
| Week52 | Q8--Q10 | 0.211 | 0.076 | 0.341 |
| Week52 | Q8--Q9 | 0.127 | 0.000 | 0.273 |
| Week52 | Q9--Q10 | 0.137 | 0.000 | 0.286 |
| Week52 | grp | 0.151 | 0.000 | 1.084 |
| Week52 | Q1 | 0.995 | 0.811 | 1.444 |
| Week52 | Q10 | 1.117 | 0.919 | 1.483 |
| Week52 | Q2 | 0.786 | 0.593 | 1.284 |
| Week52 | Q3 | 0.653 | 0.534 | 1.121 |
| Week52 | Q4 | 0.831 | 0.622 | 1.275 |
| Week52 | Q5 | 0.871 | 0.729 | 1.427 |
| Week52 | Q6 | 0.893 | 0.733 | 1.382 |
| Week52 | Q7 | 0.751 | 0.493 | 1.094 |
| Week52 | Q8 | 1.018 | 0.894 | 1.518 |
| Week52 | Q9 | 0.936 | 0.763 | 1.492 |
