# Supplementary material for: Understanding how individualised physiotherapy or advice altered different elements of disability for people with low back pain using network analysis
Source: PLoS One. 2022 Feb 10;17(2):e0263574. doi: 10.1371/journal.pone.0263574 (PMC8830646; doi:10.1371/journal.pone.0263574)
Supplement: S1 File — (ZIP) [file pone.0263574.s001.zip › supporting/sm_table3.docx]

Table S3. Centrality values

| Time | node | measure | value |
| --- | --- | --- | --- |
| Baseline | Grp | Betweenness | 0.000 |
| Baseline | Grp | Closeness | 0.418 |
| Baseline | Grp | Strength | 0.082 |
| Baseline | Q1.Pain | Betweenness | 0.467 |
| Baseline | Q1.Pain | Closeness | 0.729 |
| Baseline | Q1.Pain | Strength | 0.474 |
| Baseline | Q2.Care | Betweenness | 0.533 |
| Baseline | Q2.Care | Closeness | 0.913 |
| Baseline | Q2.Care | Strength | 0.738 |
| Baseline | Q3.Lift | Betweenness | 0.000 |
| Baseline | Q3.Lift | Closeness | 0.789 |
| Baseline | Q3.Lift | Strength | 0.490 |
| Baseline | Q4.Walk | Betweenness | 0.933 |
| Baseline | Q4.Walk | Closeness | 0.846 |
| Baseline | Q4.Walk | Strength | 0.661 |
| Baseline | Q5.Sit | Betweenness | 0.000 |
| Baseline | Q5.Sit | Closeness | 0.799 |
| Baseline | Q5.Sit | Strength | 0.416 |
| Baseline | Q6.Std | Betweenness | 0.600 |
| Baseline | Q6.Std | Closeness | 0.741 |
| Baseline | Q6.Std | Strength | 0.619 |
| Baseline | Q7.Slp | Betweenness | 0.000 |
| Baseline | Q7.Slp | Closeness | 0.501 |
| Baseline | Q7.Slp | Strength | 0.181 |
| Baseline | Q8.Life | Betweenness | 1.000 |
| Baseline | Q8.Life | Closeness | 1.000 |
| Baseline | Q8.Life | Strength | 0.759 |
| Baseline | Q9.Trav | Betweenness | 0.667 |
| Baseline | Q9.Trav | Closeness | 0.994 |
| Baseline | Q9.Trav | Strength | 0.905 |
| Baseline | Q10.Work | Betweenness | 0.467 |
| Baseline | Q10.Work | Closeness | 0.939 |
| Baseline | Q10.Work | Strength | 1.000 |
| Week 5 | Grp | Betweenness | 0.062 |
| Week 5 | Grp | Closeness | 0.618 |
| Week 5 | Grp | Strength | 0.536 |
| Week 5 | Q1.Pain | Betweenness | 0.438 |
| Week 5 | Q1.Pain | Closeness | 0.835 |
| Week 5 | Q1.Pain | Strength | 0.802 |
| Week 5 | Q2.Care | Betweenness | 0.000 |
| Week 5 | Q2.Care | Closeness | 0.731 |
| Week 5 | Q2.Care | Strength | 0.535 |
| Week 5 | Q3.Lift | Betweenness | 0.125 |
| Week 5 | Q3.Lift | Closeness | 0.702 |
| Week 5 | Q3.Lift | Strength | 0.626 |
| Week 5 | Q4.Walk | Betweenness | 0.000 |
| Week 5 | Q4.Walk | Closeness | 0.596 |
| Week 5 | Q4.Walk | Strength | 0.596 |
| Week 5 | Q5.Sit | Betweenness | 0.000 |
| Week 5 | Q5.Sit | Closeness | 0.701 |
| Week 5 | Q5.Sit | Strength | 0.497 |
| Week 5 | Q6.Std | Betweenness | 0.000 |
| Week 5 | Q6.Std | Closeness | 0.680 |
| Week 5 | Q6.Std | Strength | 0.508 |
| Week 5 | Q7.Slp | Betweenness | 0.125 |
| Week 5 | Q7.Slp | Closeness | 0.742 |
| Week 5 | Q7.Slp | Strength | 0.655 |
| Week 5 | Q8.Life | Betweenness | 1.000 |
| Week 5 | Q8.Life | Closeness | 1.000 |
| Week 5 | Q8.Life | Strength | 1.000 |
| Week 5 | Q9.Trav | Betweenness | 0.500 |
| Week 5 | Q9.Trav | Closeness | 0.840 |
| Week 5 | Q9.Trav | Strength | 0.926 |
| Week 5 | Q10.Work | Betweenness | 0.562 |
| Week 5 | Q10.Work | Closeness | 0.896 |
| Week 5 | Q10.Work | Strength | 0.887 |
| Week 10 | Grp | Betweenness | 0.000 |
| Week 10 | Grp | Closeness | 0.593 |
| Week 10 | Grp | Strength | 0.306 |
| Week 10 | Q1.Pain | Betweenness | 0.750 |
| Week 10 | Q1.Pain | Closeness | 0.897 |
| Week 10 | Q1.Pain | Strength | 0.889 |
| Week 10 | Q2.Care | Betweenness | 0.000 |
| Week 10 | Q2.Care | Closeness | 0.713 |
| Week 10 | Q2.Care | Strength | 0.514 |
| Week 10 | Q3.Lift | Betweenness | 0.000 |
| Week 10 | Q3.Lift | Closeness | 0.816 |
| Week 10 | Q3.Lift | Strength | 0.653 |
| Week 10 | Q4.Walk | Betweenness | 0.125 |
| Week 10 | Q4.Walk | Closeness | 0.721 |
| Week 10 | Q4.Walk | Strength | 0.514 |
| Week 10 | Q5.Sit | Betweenness | 0.250 |
| Week 10 | Q5.Sit | Closeness | 0.870 |
| Week 10 | Q5.Sit | Strength | 0.598 |
| Week 10 | Q6.Std | Betweenness | 0.625 |
| Week 10 | Q6.Std | Closeness | 0.850 |
| Week 10 | Q6.Std | Strength | 0.749 |
| Week 10 | Q7.Slp | Betweenness | 0.625 |
| Week 10 | Q7.Slp | Closeness | 0.779 |
| Week 10 | Q7.Slp | Strength | 0.552 |
| Week 10 | Q8.Life | Betweenness | 0.375 |
| Week 10 | Q8.Life | Closeness | 0.903 |
| Week 10 | Q8.Life | Strength | 0.774 |
| Week 10 | Q9.Trav | Betweenness | 0.375 |
| Week 10 | Q9.Trav | Closeness | 0.892 |
| Week 10 | Q9.Trav | Strength | 0.800 |
| Week 10 | Q10.Work | Betweenness | 1.000 |
| Week 10 | Q10.Work | Closeness | 1.000 |
| Week 10 | Q10.Work | Strength | 1.000 |
| Week 26 | Grp | Betweenness | 0.000 |
| Week 26 | Grp | Closeness | 0.629 |
| Week 26 | Grp | Strength | 0.265 |
| Week 26 | Q1.Pain | Betweenness | 1.000 |
| Week 26 | Q1.Pain | Closeness | 1.000 |
| Week 26 | Q1.Pain | Strength | 0.945 |
| Week 26 | Q2.Care | Betweenness | 0.077 |
| Week 26 | Q2.Care | Closeness | 0.776 |
| Week 26 | Q2.Care | Strength | 0.534 |
| Week 26 | Q3.Lift | Betweenness | 0.000 |
| Week 26 | Q3.Lift | Closeness | 0.675 |
| Week 26 | Q3.Lift | Strength | 0.541 |
| Week 26 | Q4.Walk | Betweenness | 0.231 |
| Week 26 | Q4.Walk | Closeness | 0.755 |
| Week 26 | Q4.Walk | Strength | 0.682 |
| Week 26 | Q5.Sit | Betweenness | 0.308 |
| Week 26 | Q5.Sit | Closeness | 0.862 |
| Week 26 | Q5.Sit | Strength | 0.769 |
| Week 26 | Q6.Std | Betweenness | 0.154 |
| Week 26 | Q6.Std | Closeness | 0.787 |
| Week 26 | Q6.Std | Strength | 0.683 |
| Week 26 | Q7.Slp | Betweenness | 0.231 |
| Week 26 | Q7.Slp | Closeness | 0.785 |
| Week 26 | Q7.Slp | Strength | 0.645 |
| Week 26 | Q8.Life | Betweenness | 0.154 |
| Week 26 | Q8.Life | Closeness | 0.860 |
| Week 26 | Q8.Life | Strength | 0.794 |
| Week 26 | Q9.Trav | Betweenness | 0.154 |
| Week 26 | Q9.Trav | Closeness | 0.823 |
| Week 26 | Q9.Trav | Strength | 0.837 |
| Week 26 | Q10.Work | Betweenness | 0.615 |
| Week 26 | Q10.Work | Closeness | 0.973 |
| Week 26 | Q10.Work | Strength | 1.000 |
| Week52 | Grp | Betweenness | 0.000 |
| Week52 | Grp | Closeness | 0.439 |
| Week52 | Grp | Strength | 0.135 |
| Week52 | Q1.Pain | Betweenness | 0.375 |
| Week52 | Q1.Pain | Closeness | 0.870 |
| Week52 | Q1.Pain | Strength | 0.890 |
| Week52 | Q2.Care | Betweenness | 0.250 |
| Week52 | Q2.Care | Closeness | 0.974 |
| Week52 | Q2.Care | Strength | 0.704 |
| Week52 | Q3.Lift | Betweenness | 0.000 |
| Week52 | Q3.Lift | Closeness | 0.761 |
| Week52 | Q3.Lift | Strength | 0.585 |
| Week52 | Q4.Walk | Betweenness | 0.000 |
| Week52 | Q4.Walk | Closeness | 0.817 |
| Week52 | Q4.Walk | Strength | 0.743 |
| Week52 | Q5.Sit | Betweenness | 0.375 |
| Week52 | Q5.Sit | Closeness | 0.873 |
| Week52 | Q5.Sit | Strength | 0.779 |
| Week52 | Q6.Std | Betweenness | 0.375 |
| Week52 | Q6.Std | Closeness | 0.896 |
| Week52 | Q6.Std | Strength | 0.799 |
| Week52 | Q7.Slp | Betweenness | 0.875 |
| Week52 | Q7.Slp | Closeness | 0.750 |
| Week52 | Q7.Slp | Strength | 0.672 |
| Week52 | Q8.Life | Betweenness | 1.000 |
| Week52 | Q8.Life | Closeness | 1.000 |
| Week52 | Q8.Life | Strength | 0.912 |
| Week52 | Q9.Trav | Betweenness | 0.125 |
| Week52 | Q9.Trav | Closeness | 0.957 |
| Week52 | Q9.Trav | Strength | 0.838 |
| Week52 | Q10.Work | Betweenness | 1.000 |
| Week52 | Q10.Work | Closeness | 0.949 |
| Week52 | Q10.Work | Strength | 1.000 |
