# Supplementary figures and images for: Understanding how individualised physiotherapy or advice altered different elements of disability for people with low back pain using network analysis
Source: PLoS One. 2022 Feb 10;17(2):e0263574. doi: 10.1371/journal.pone.0263574 (PMC8830646; doi:10.1371/journal.pone.0263574)

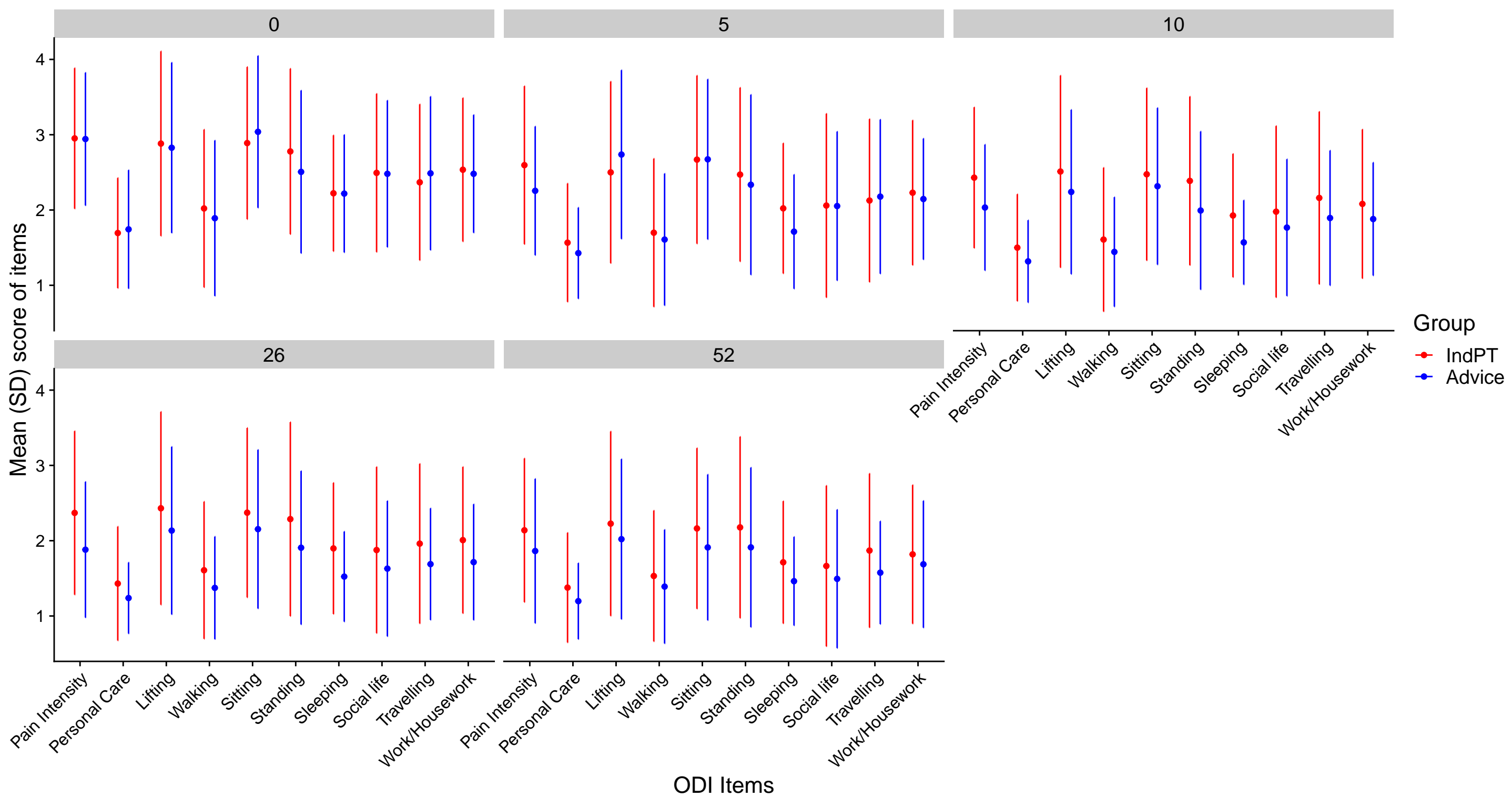

Supplement: S1 File — (ZIP) [file pone.0263574.s001.zip › supporting/s1_fig.pdf]
